# Corticosteroid use and intensive care unit-acquired weakness: a systematic review and meta-analysis

**DOI:** 10.1186/s13054-018-2111-0

**Published:** 2018-08-03

**Authors:** Tao Yang, Zhiqiang Li, Li Jiang, Xiuming Xi

**Affiliations:** 1 Department of Critical Care Medicine, Fu Xing Hospital, Capital Medical University, 20A Fu Xing Men Wai Da Jie, Xicheng District, Beijing, 100038 China; 2 Department of Critical Care Medicine, North China University of Science and Technology Affiliated Hospital, Tangshan, China

**Keywords:** Intensive care unit, ICU-acquired weakness, Corticosteroids, Corticosteroid use, Corticosteroid therapy, Systematic review

## Abstract

**Background:**

The association between corticosteroid use and intensive care unit (ICU)-acquired weakness remains unclear. We evaluated the relationship between corticosteroid use and ICU-acquired weakness in critically ill adult patients.

**Methods:**

The PubMed, Embase, Web of Science, Cochrane Central Register of Controlled Trials, and Cumulative Index of Nursing and Allied Health Literature databases were searched from database inception until October 10, 2017. Two authors independently screened the titles/abstracts and reviewed full-text articles. Randomized controlled trials and prospective cohort studies evaluating the association between corticosteroids and ICU-acquired weakness in adult ICU patients were selected. Data extraction from the included studies was accomplished by two independent reviewers. Meta-analysis was performed using Stata version 12.0. The results were analyzed using odds ratios (ORs) and 95% confidence intervals (CIs). Data were pooled using a random effects model, and heterogeneity was evaluated using the χ^2^ and *I*^2^ statistics. Publication bias was qualitatively analyzed with funnel plots, and quantitatively analyzed with Begg’s test and Egger’s test.

**Results:**

One randomized controlled trial and 17 prospective cohort studies were included in this review. After a meta-analysis, the effect sizes of the included studies indicated a statistically significant association between corticosteroid use and ICU-acquired weakness (OR 1.84; 95% CI 1.26–2.67; *I*^2^ = 67.2%). Subgroup analyses suggested a significant association between corticosteroid use and studies limited to patients with clinical weakness (OR 2.06; 95% CI 1.27–3.33; *I*^2^ = 60.6%), patients with mechanical ventilation (OR 2.00; 95% CI 1.23–3.27; *I*^2^ = 66.0%), and a large sample size (OR 1.61; 95% CI 1.02–2.53; *I*^2^ = 74.9%), and not studies limited to patients with abnormal electrophysiology (OR 1.65; 95% CI 0.92–2.95; *I*^2^ = 70.6%) or patients with sepsis (OR 1.96; 95% CI 0.61–6.30; *I*^2^ = 80.8%); however, statistical heterogeneity was obvious. No significant publication biases were found in the review. The overall quality of the evidence was high for the randomized controlled trial and very low for the included prospective cohort studies.

**Conclusions:**

The review suggested a significant association between corticosteroid use and ICU-acquired weakness. Thus, exposure to corticosteroids should be limited, or the administration time should be shortened in clinical practice to reduce the risk of ICU-acquired weakness.

**Electronic supplementary material:**

The online version of this article (10.1186/s13054-018-2111-0) contains supplementary material, which is available to authorized users.

## Background

Intensive care unit-acquired weakness (ICUAW) is a common neuromuscular complication of critical illness. ICUAW is associated with delayed weaning, longer intensive care unit (ICU) and hospital stays, increased healthcare-related costs, and higher ICU-related and hospitalization-related mortality [1–3]. Corticosteroid therapy is still the key treatment and recommendation for specific critically ill patients [4, 5] because of its strong anti-inflammatory and anti-fibrotic effects. Corticosteroid therapy results in a shorter duration of mechanical ventilation, a faster resolution of shock [6], more vasopressor-free and organ-failure-free days [7], and lower mortality [7–9] in patients with refractory septic shock. For patients with acute respiratory distress syndrome (ARDS), corticosteroid therapy may also improve hypoxemia [10] and reduce the duration of mechanical ventilation [11, 12] and the ICU hospitalization period [13]. ICUAW occurs commonly in critically ill patients, but the role of corticosteroid therapy in ICUAW remains controversial. Researchers and authors have raised significant concerns regarding the side effects of corticosteroids in terms of ICUAW development and have attempted to examine the relationship. Some clinical studies have indicated that corticosteroids may contribute to developing ICUAW, yet others have demonstrated decreasing odds of developing ICUAW. However, other studies could not identify the effect of corticosteroids on ICUAW. In this review, we provide a meta-analysis of randomized controlled trials (RCTs) and prospective cohort studies to assess the association between corticosteroid use and ICUAW development.

No universal recommendation or consensus on the definition or classification of the disease exists; after consulting the literature [14], the relatively broad term “intensive care unit-acquired weakness (ICUAW)” was selected for use in this review. Although there was no diagnostic gold standard for ICUAW, three diagnostic methods were recommended to identify ICUAW [14, 15]: manual muscle testing (Medical Research Council (MRC) weakness scale), electrophysiological studies, and the histopathology of muscle or nerve tissue. However, muscle or nerve tissue biopsy was rarely used in the studies. This review explores the adverse effect of corticosteroids on ICUAW development, from patients with clinical weakness to patients with clinically undetectable neuromuscular dysfunction.

## Methods

This study was conducted according to the Preferred Reporting Items for Systematic Reviews and Meta-Analyses: the PRISMA statement [16].

### Search strategy

A systematic literature review of all of the pertinent English language studies was undertaken in the following databases from inception through October 10, 2017: PubMed, Embase, Cochrane Central Register of Controlled Trials, Web of Science, and Cumulative Index of Nursing and Allied Health Literature. The search terms were used for PubMed (Additional file 1) and the other databases. In addition, a manual search of references cited by the selected articles and relevant review articles was performed to identify other eligible studies.

### Selection criteria

All studies satisfying the following criteria were included: age > 18; RCTs and prospective cohort studies; diagnoses of ICUAW confirmed using manual muscle testing (MRC weakness scale) or diagnostic tests (electrophysiological studies, histopathology of muscle or nerve tissue); and studies that evaluated the use of corticosteroids and incidence of ICUAW. The exclusion criteria were as follows: patients with primary myopathies (e.g., idiopathic inflammatory myopathies) or polyneuropathies (e.g., myasthenia gravis, Guillain-Barré syndrome); and studies with insufficient data reported.

### Study selection and data abstraction

Two reviewers (TY and ZqL) independently reviewed and selected studies based on the inclusion criteria. Data were extracted independently by each reviewer using a standardized data collection form. The following data were collected from each study: author information, publication year, study design, study location, inclusion and exclusion criteria, tools of neuromuscular evaluation, number of participants, ICUAW incidence, and number of ICUAW patients who were given and not given corticosteroids. Disagreements in study selection or data extraction were resolved by either consensus or a third-party decision. Authors of the included studies were contacted when data required clarification.

### Study quality assessment

Two reviewers (TY and ZqL) independently assessed the methodological quality of each study using the Newcastle–Ottawa scale [17] for prospective studies and the Cochrane Collaboration tool [17] for RCTs.

### Data analysis

Meta-analysis was performed using Stata version 12.0 (StataCorp, College Station, TX, USA), and the results were analyzed using odds ratios (ORs) and 95% confidence intervals (CIs). Data were pooled using the DerSimonian and Laird random effects model. Heterogeneity was assessed using the χ^2^ statistic with *P* ≤ 0.1 considered statistically significant. The impact of statistical heterogeneity on the study results was estimated by calculating the *I*^2^ statistic. Values of the *I*^2^ statistic above 50% were regarded as a cutoff point for considerable heterogeneity. Subgroup analyses examined: RCT and prospective cohort studies; studies using clinical muscle testing and electrophysiology as a diagnostic method; studies using mechanical and nonmechanical ventilation as inclusion criteria; studies using sepsis and nonsepsis as inclusion criteria; and studies with relatively large (*n* ≥ 100) and small (*n* < 100) sample sizes. Publication bias was examined using funnel plots for qualitative assessment, using Begg’s rank correlation test and Egger’s linear regression test for quantitative assessment.

### Summary of findings

The Grading of Recommendations, Assessment, Development and Evaluation (GRADE) assessment method was employed to determine the quality of evidence in our review associated with the main outcome (incidence of ICUAW). Two reviewers (TY and ZqL) independently graded the evidence prior to agreement and created the ‘Summary of findings’ table using GRADE software [17]. We considered risk of bias, directness of evidence, heterogeneity of the data, precision of effect, and risk of publication bias as the factors influencing assessment of the review.

## Results

### Study search and selection

**Fig. 1:**
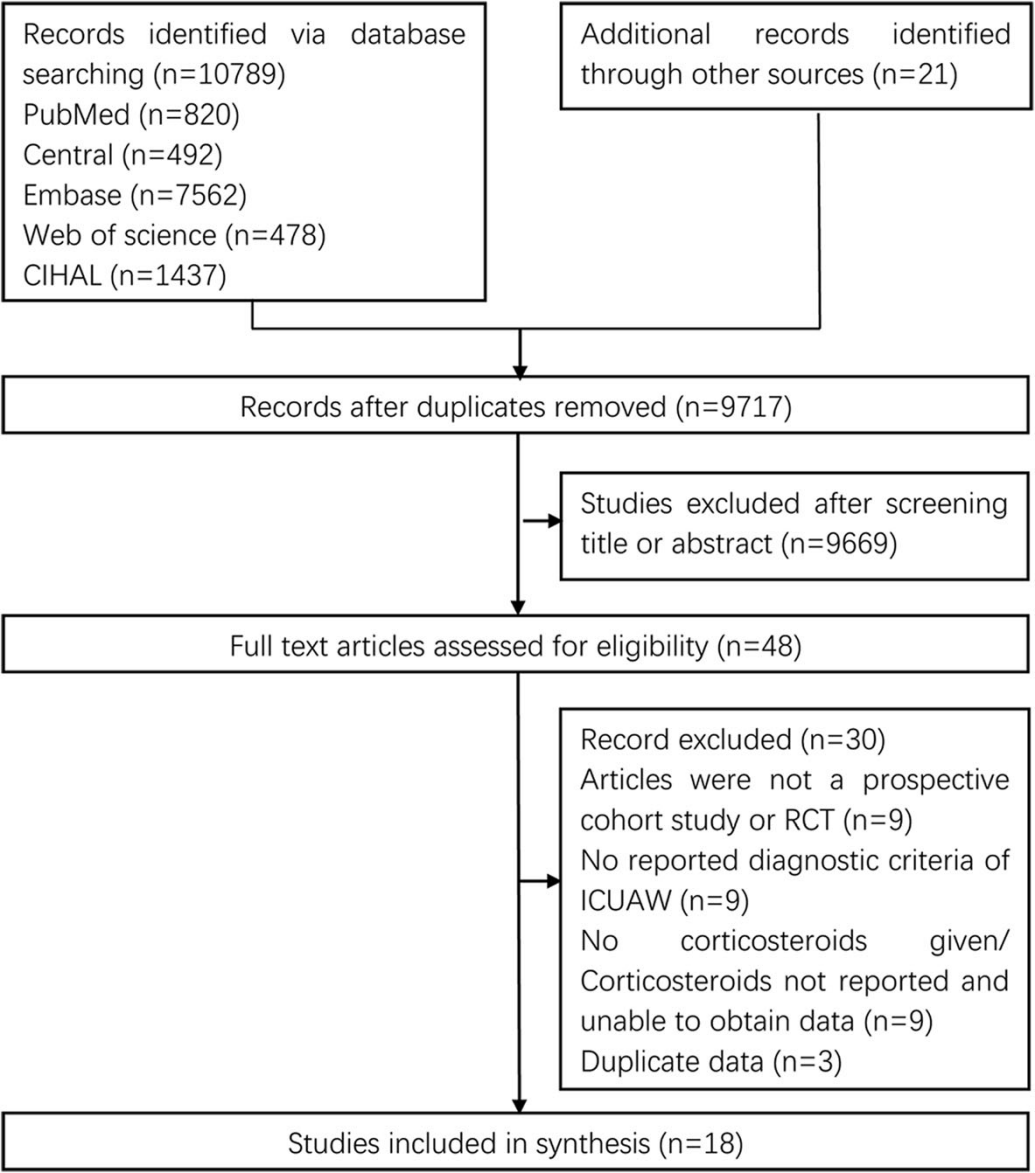
Flow diagram of the study selection process. CIHAL Cumulative Index of Nursing and Allied Health Literature, ICUAW intensive care unit-acquired weakness, RCT randomized controlled trial

The electronic search yielded a total of 10,789 citations (Fig. 1). Twenty-one additional articles were identified through other sources. After screening the titles and abstracts, 48 articles were selected for full-text review. Thirty articles did not meet the inclusion criteria and were excluded, and therefore 18 studies were included in this review.

### Study characteristics and quality

The characteristics of the included studies in this systematic review are presented in Table 1. They included one RCT [18] and 17 prospective cohort studies [1, 19–34]. The number of participants in each study ranged from 20 to 412. The studies were carried out in the United States [21, 27], India [19], Vietnam [1], Belgium [28], the Netherlands [20, 33], Germany [18, 25], Switzerland [24], France [23, 29, 31], Spain [32], Greece [22, 26], Canada [30], and England [34]. Diagnosis of ICUAW was accomplished in eight studies [18, 20, 21, 23, 24, 26, 27, 31] using the MRC scale and in 10 studies [1, 19, 22, 25, 28–30, 32–34] using electrophysiological evaluation. Participant inclusion criteria included mechanical ventilation in 12 studies [20, 21, 23–25, 27–29, 31–34], systemic inflammatory response syndrome (SIRS) or sepsis in five studies [18, 19, 24, 30, 32], and length of ICU stay in four studies [1, 22, 26, 34]. ICU mortality differed across the studies.

**Table 1:** Characteristics of the selected studies

Study	Study design	Country	Setting	Population	*n*	Examination	ICUAW	Use of CS ^a^	ICU mortality (%)^a^
Keh et al., 2016 [18]	RCT	Germany	MSICU	Severe sepsis	375	Clinical	82	46 vs 140	NR
Gupta and Mishra, 2016 [19]	Prospective cohort	India	MICU	Sepsis	100	EMG	37	26 vs 12	NR
Nguyen The and Nguyen Huu, 2015 [1]	Prospective cohort	Vietnam	MSICU	ICU LOS ≥ 10 days	133	EMG	73	29 vs 18	49% vs 30%
Patel et al., 2014 [21]	Prospective cohort	America	MICU	MV ≥ 24 h	104	Clinical	41	35 vs 44	NR
Wieske et al., 2014 [20]	Prospective cohort	Netherlands	MSICU	MV ≥ 2 days	212	Clinical	103	81 vs 63	34% vs 9%
Anastasopoulos et al., 2011 [22]	Prospective cohort	Greece	MSICU	ICU LOS ≥ 7 days	190	EMG	40	28 vs 102	32.5% vs NR
Sharshar et al., 2010 [23]	Prospective cohort	France	MICU, SICU	MV > 7 days	86	Clinical	39	29 vs 22	NR
Brunello et al., 2010 [24]	Prospective cohort	Switzerland	MSICU	MV > 48 h and SIRS	39	Clinical	13	4 vs 0	62% vs 23%
Weber-Carstens et al., 2009 [25]	Prospective cohort	Germany	SICU	MV and SAPS II ≥ 20	56	EMG	34	21 vs 5	NR
Nanas et al., 2008 [26]	Prospective cohort	Greece	MSICU	LOS > 10 days	185	Clinical	44	7 vs 31	36% vs 20%
Ali et al., 2008 [27]	Prospective cohort	America	MICU, NICU	MV > 5days	136	Clinical	35	16 vs 42	31.4% vs 6%
Hermans et al., 2007 [28]	Prospective cohort	Belgium	MICU	MV > 7 days	412	EMG	188	123 vs 156	NR
Khan et al., 2006 [30]	Prospective cohort	Canada	MSICU	Sepsis	20	EMG	10	4 vs 4	55% vs 0%
Lefaucheur et al., 2006 [29]	Prospective cohort	France	MICU	MV > 7 days, diffuse weakness	30	EMG	26	15 vs 1	NR
De Jonghe et al., 2002 [31]	Prospective cohort	France	MICU, SICU	MV > 7 days and awake	95	Clinical	24	13 vs 13	17% vs 6%
de Letter et al., 2001 [33]	Prospective cohort	Netherlands	MSICU	MV ≥ 4 days	97	EMG	34	9 vs 18	NR
Garnacho-Montero et al., 2001 [32]	Prospective cohort	Spain	MSICU	MV > 10 days and sepsis with MOF	73	EMG	50	7 vs 4	66% vs 52%
Coakley et al., 1998 [34]	Prospective cohort	England	MSICU	MV and ICU LOS > 7 days	44	EMG	37	11 vs 2	NR

*ICUAW* intensive care unit-acquired weakness, *CS* corticosteroids, *ICU* intensive care unit, *RCT* randomized controlled trial, *MICU* medical ICU, *MSICU* medical–surgical ICU, *SICU* surgical ICU, *NR* not reported, *EMG* electromyography, *LOS* length of stay, *MV* mechanical ventilation, *SIRS* systemic inflammatory response syndrome, *SAPS* Simplified Acute Physiology Score, *MOF* multiple organ failure

^a^Comparison between ICUAW and no ICUAW

The methodological quality assessment of the included reports is presented in Table 2. The risk of bias of the randomized trial was low, and the overall risk of bias of the prospective studies was acceptable in general. Three of the 17 observational studies made statistical comparisons with multivariable regression analysis for corticosteroids, and therefore the other 14 studies received no scores for comparability. Four studies did not report whether the assessments were independently blinded for clinicians or physical therapists.

**Table 2:** Methodology and reporting assessment

Cochrane Collaboration tool for assessing risk of bias									
Study	Sequence generation	Allocation concealment	Blinding of participants, personnel, and outcome assessors		Incomplete outcome data	Selective outcome reporting	Other potential threats to validity		Risk of bias
Keh et al., 2016 [18]	Y	Y	Y		Y	Y	Y		Low
Newcastle–Ottawa quality assessment scale for cohort studies									
Study	Selection				Comparability	Outcome			Score
	Exposed representative?	Nonexposed representative?	Ascertainment of exposure	Outcome of interest not present at start		Assessment of outcome	Adequate duration of follow-up	Completeness of follow-up	
Gupta and Mishra, 2016 [19]	Y	Y	Y	Y	Y, Y	Y	Y	Y	9
Nguyen The and Nguyen Huu, 2015 [1]	Y	Y	Y	Y	N, N	Y	Y	Y	7
Wieskeet al., 2014 [20]	Y	Y	Y	Y	N, N	Y	Y	Y	7
Patel et al., 2014 [21]	Y	Y	Y	Y	N, N	Y	Y	Y	7
Anastasopoulos et al., 2011 [22]	Y	Y	Y	Y	N, N	Y	Y	Y	7
Brunello et al., 2010 [24]	Y	Y	Y	Y	Y, Y	N	Y	N	7
Sharshar et al., 2010 [23]	Y	Y	Y	Y	N, N	N	Y	Y	6
Weber-Carstens et al., 2009 [25]	Y	Y	Y	Y	N, N	Y	Y	Y	7
Nanas et al., 2008 [26]	Y	Y	Y	Y	N, N	N	Y	Y	6
Ali et al., 2008 [27]	Y	Y	Y	Y	N, N	Y	Y	Y	7
Hermans et al., 2007 [28]	Y	Y	Y	Y	N, N	Y	Y	Y	7
Khan et al., 2006 [30]	Y	Y	Y	Y	N, N	Y	Y	N	6
Lefaucheur et al., 2006 [29]	Y	Y	Y	Y	N, N	Y	Y	Y	7
De Jonghe et al., 2002 [31]	Y	Y	Y	Y	Y, Y	Y	Y	Y	9
de Letter et al., 2001 [33]	Y	Y	Y	Y	N, N	N	Y	Y	6
Garnacho-Montero et al., 2001 [32]	Y	Y	Y	Y	N, N	Y	Y	Y	7
Coakley et al., 1998 [34]	Y	Y	Y	Y	N, N	Y	Y	Y	7

*Y* criteria satisfied, *N* criteria not satisfied

### Corticosteroids and ICUAW

**Fig. 2:**
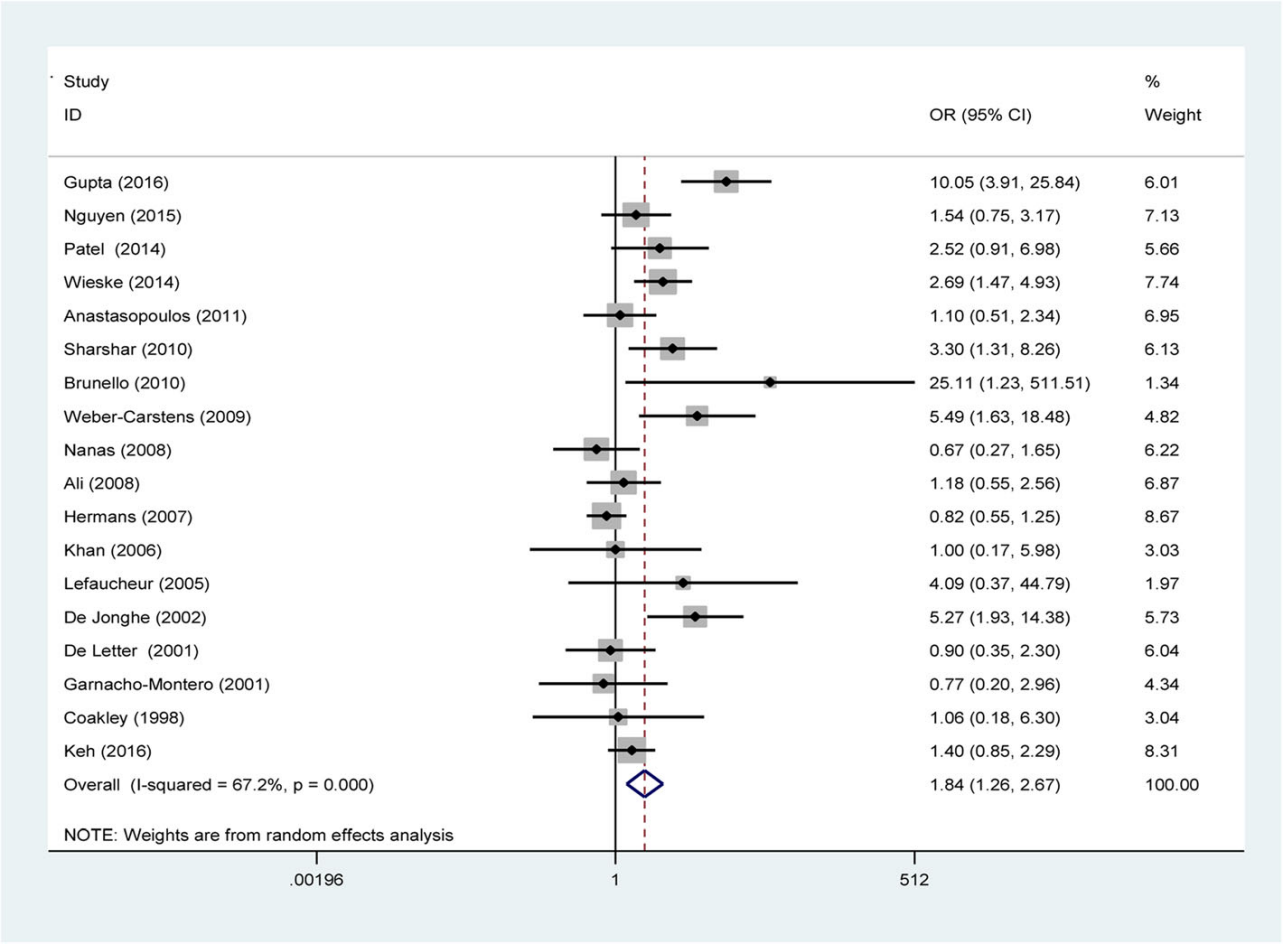
Forest plot of associations between corticosteroid use and ICUAW. CI confidence interval, OR odds ratio

When the 18 studies were pooled together (Fig. 2), the effect size analysis (OR 1.84; 95% CI 1.26–2.67; *P* = 0.002) indicated that the use of corticosteroids was significantly associated with increased odds of developing ICUAW. Data were pooled using a random effects model considering the observed heterogeneity (τ^2^ = 0.38; χ^2^ = 51.87, df = 17 (*P* < 0.001); *I*^2^ = 67.2%). The overall incidence of ICUAW was 43% in the corticosteroid group versus 34% in the control group.

### Subgroup analyses

#### RCTs versus prospective cohort studies

The subgroup analyses are presented in Table 3. One RCT revealed no significant association between corticosteroids and ICUAW (OR 1.40; 95% CI 0.85–2.29; *P* = 0.184), and the GRADE quality of evidence was high for this trial (Additional file 2). The meta-analysis of 17 prospective cohort studies (OR 1.90; 95% CI 1.25–2.89; *P* = 0.003) showed a significant association with a random effects model considering the observed heterogeneity (τ^2^ = 0.46; χ^2^ = 51.66, df = 16 (*P* < 0.001); *I*^2^ = 69.0%). The incidence of ICUAW was 46% in the corticosteroid group versus 36% in the control group; however, the GRADE quality of evidence was very low (Additional file 2). There was no significantly statistical heterogeneity found between the subgroups based on a test of interaction (*P* = 0.35).

**Table 3:** Subgroup analyses

Analysis	Study	*n*	*I*^2^ (%)	Ph	OR	95% CI	Pe	Pi	Incidence corticosteroid (%)	Incidence control (%)
Study type										
RCT	[18]	375			1.40	0.85–2.29	0.184		25	19
Prospective cohort studies	[1, 19–34]	2012	69.0	< 0.001	1.90	1.25–2.89	0.003	0.35	46	36
Diagnostic method										
Clinical assessment	[18, 20, 21, 23, 24, 26, 27, 31]	1232	60.6	0.013	2.06	1.27–3.33	0.003		39	23
Electrophysiology	[1, 19, 22, 25, 28–30, 32–34]	1155	70.6	< 0.001	1.65	0.92–2.95	0.093	0.56	46	46
Inclusion criterion										
Sepsis	[18, 19, 30, 32]	568	80.8	0.001	1.96	0.61–6.30	0.260		34	30
Nonsepsis	[1, 20–29, 31, 33, 34]	1819	63.0	0.001	1.77	1.18–2.64	0.006	0.87	45	35
MV	[20, 21, 23–25, 27–29, 31–34]	1384	66.0	0.001	2.00	1.23–3.27	0.006		50	40
Non-MV	[1, 18, 19, 22, 26, 30]	1003	74.4	0.002	1.61	0.83–3.13	0.161	0.61	31	26
Sample size										
n ≥ 100	[1, 18–22, 26–28]	1847	74.9	< 0.001	1.62	1.02–2.53	0.042		39	30
*n* < 100	[23–25, 29–34]	540	49.3	0.046	2.32	1.21–4.42	0.011	0.36	62	43

I^*2*^ *I*-squared statistic test for heterogeneity, *Ph P* value for test of heterogeneity, *OR* odds ratio, *CI* confidence interval, *Pe P* value for the effect estimate for each subgroup, *Pi P* value for interaction tests of heterogeneity between subgroups, *RCT* randomized controlled trial, *MV* mechanical ventilation

#### Clinical assessment versus electrophysiology

Eight studies [18, 20, 21, 23, 24, 26, 27, 31] examined the association between the use of corticosteroids and patients with clinical weakness and demonstrated an incidence of 39% in the corticosteroid group and 23% in the control group. The overall effect size (OR 2.06; 95% CI 1.27–3.33; *P* = 0.003) demonstrated a significant association with a random effects model considering the observed heterogeneity (τ^2^ = 0.27; χ^2^ = 17.78, df = 7 (*P* = 0.013); *I*^2^ = 60.6%). Ten observational studies [1, 19, 22, 25, 28–30, 32–34] reported an association between the use of corticosteroids and patients with abnormal electrophysiology and showed an event rate of 46% in the corticosteroid group and 46% in the control group. The pooled effect size (OR 1.65; 95% CI 0.92–2.95; *P* = 0.093) revealed no significant association. Data were pooled using a random effects model considering the observed heterogeneity (τ^2^ = 0.53; χ^2^ = 30.63, df = 9 (*P* < 0.001); *I*^2^ = 70.6%). No statistically significant heterogeneity between the subgroups was found based on a test of the interaction (*P* = 0.56).

#### Sepsis versus nonsepsis

Four trials [18, 19, 30, 32] with sepsis as the inclusion criterion reported an association between the use of corticosteroids and ICUAW, and demonstrated an incidence of 34% in the corticosteroid group and 30% in the control group. The pooled effect size (OR 1.96; 95% CI 0.61–6.30; *P* = 0.260) revealed no significant association. Data were pooled using a random effects model considering the observed heterogeneity (τ^2^ = 1.08; χ^2^ = 15.65, df = 3 (*P* = 0.001); *I*^2^ = 80.8%). The remaining 14 studies [1, 20–29, 31, 33, 34] without sepsis as an inclusion criterion showed an unadjusted event rate in the corticosteroid group of 45% versus 35% in the control group. The pooled effect size (OR 1.77; 95% CI 1.18–2.64; *P* = 0.006) demonstrated a significant association with a random effects model considering the observed heterogeneity (τ^2^ = 0.32; χ^2^ = 35.18, df = 13 (*P* = 0.001); *I*^2^ = 63.0%). No statistically significant heterogeneity between the subgroups was found based on a test of the interaction (*P* = 0.87).

#### Mechanical ventilation versus nonmechanical ventilation

Twelve observational studies [20, 21, 23–25, 27–29, 31–34] using mechanical ventilation as an inclusion criterion examined the association between the use of corticosteroids and ICUAW, and showed an event rate of 50% in the corticosteroid group and 40% in the control group. The overall effect size (OR 2.00; 95% CI 1.23–3.27; *P* = 0.006) demonstrated a significant association with a random effects model considering the observed heterogeneity (τ^2^ = 0.42; χ^2^ = 32.32, df = 11 (*P* = 0.001); *I*^2^ = 66.0%). The remaining six studies [1, 18, 19, 22, 26, 30] without mechanical ventilation as an inclusion criterion showed an unadjusted event rate in the corticosteroid group of 31% versus 26% in the control group. The pooled effect size (OR 1.61; 95% CI 0.83–3.13; *P* = 0.161) revealed no significant association with a random effects model considering the observed heterogeneity (τ^2^ = 0.48; χ^2^ = 19.54, df = 5 (*P* = 0.002); *I*^2^ = 74.4%). No statistically significant heterogeneity between the subgroups was found based on a test of the interaction (*P* = 0.61).

#### Sample sizes (*n* ≥ 100 versus *n* < 100)

After the results of the nine studies [1, 18–22, 26–28] with sample sizes greater than 100 were incorporated, the pooled effect size (OR 1.62; 95% CI 1.02–2.53; *P* = 0.042) still demonstrated a significant association between corticosteroid use and ICUAW with a random effects model considering the observed heterogeneity (τ^2^ = 0.35; χ^2^ = 31.92, df = 8 (*P* < 0.001); *I*^2^ = 74.9%), with an event rate of 39% in the corticosteroid group and 30% in the control group. The remaining nine studies [23–25, 29–34] with relatively small sample sizes (*n* < 100) showed an unadjusted event rate in the corticosteroid group of 62% versus 43% in the control group. The pooled effect size (OR 2.32; 95% CI 1.21–4.42; *P* = 0.011) demonstrated a significant association with a random effects model considering the observed heterogeneity (τ^2^ = 0.44; χ^2^ = 15.77, df = 8 (*P* = 0.046); *I*^2^ = 49.3%). No statistically significant heterogeneity between the subgroups was found based on a test of the interaction (*P* = 0.36).

### Heterogeneity

#### Methodological heterogeneity

Methodological heterogeneity was found among the included studies. Two study design types were utilized among the included studies, and two diagnostic methods were used in the included studies. Sample sizes differed across the included studies; small and large studies were delineated by a cutoff value of 100 subjects. This methodological heterogeneity led to three comparisons in the review: RCTs versus prospective cohort studies, clinical assessment versus electrophysiology, and sample size analysis (*n* ≥ 100 versus *n* < 100).

#### Clinical heterogeneity

Clinical heterogeneity was also observed in the included studies. The study cohorts were differed due to different inclusion criteria among the included studies, which led to two comparisons in the review: sepsis versus nonsepsis, and mechanical ventilation versus nonmechanical ventilation.

#### Statistical heterogeneity

There were high levels of statistical heterogeneity in the review, and statistical heterogeneity remained substantial within each of the five comparisons described (presented in Table 3).

### Assessment of publication biases

**Fig. 3:**
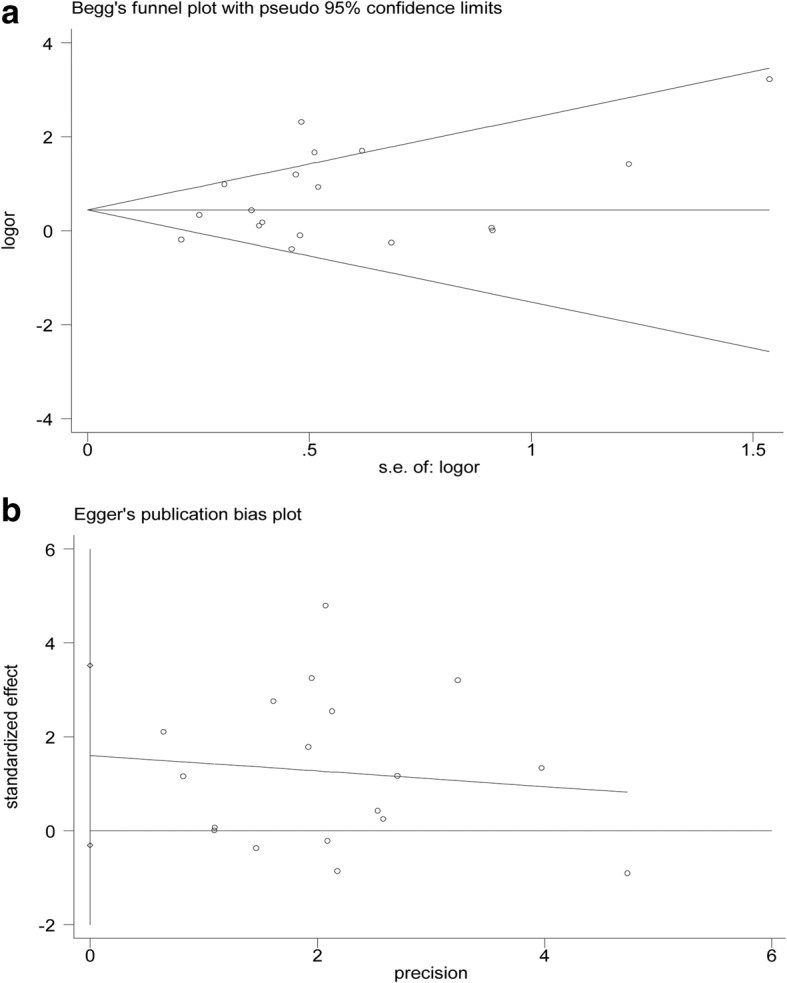
Funnel plots. **a** Begg’s funnel plots of included studies. **b** Egger’s funnel plots of included studies. s.e. standard error

Funnel plots were used to estimate the publication bias. As depicted in Fig. 3a, b, there was no significant asymmetry found in the funnel plots. Begg’s test (*z* = 1.06, *P* = 0.289) and Egger’s test (*t* = 1.77, *P* = 0.095) were adopted to detect publication bias in the meta-analysis, and no significant biases were found.

## Discussion

This review synthesized data on the relationship between corticosteroids and ICUAW. We identified 18 studies with a total of 2387 enrolled patients. When the studies were pooled together, the effect size analysis showed that corticosteroid use was a significant risk for developing ICUAW.

Corticosteroid therapy was still an essential treatment option in selected critically ill patients, such as those with refractory septic shock and ARDS. Similar muscle changes to those of animals as a result of corticosteroid therapy had been found in ICU patients [35]. Corticosteroid therapy was found to cause changes in specific gene expression to indicate the inhibition of protein synthesis resulting in promoting muscle wasting [36, 37]. Evaluating the effect of corticosteroid therapy on ICUAW development is critical. Thus, this systematic review synthesized data on the relationship between the use of corticosteroids and ICUAW in ICU patients. In addition, the effect of corticosteroid therapy on ICUAW is complex and may also depend on the duration and cumulative dosage of the corticosteroids. Of the included studies, duration of the corticosteroids was not found to be an independent risk factor for ICUAW [28, 31], but the cumulative doses of corticosteroids were significantly higher in patients with ICUAW than in those without ICUAW in two studies [23, 25] based on univariate analysis. Thus, exposure to corticosteroids should be limited or the dose lowered in clinical practice to reduce the risk of ICUAW.

Our subgroup analyses revealed a stronger association in patients with clinical weakness but not in patients with abnormal electrophysiology. The use of corticosteroids was found to be significantly associated with muscle weakness in the review. However, within the electrophysiology subgroup, the incidences of ICUAW in the corticosteroid and control groups were higher than those found in the clinical assessment subgroup. ICUAW is essentially a clinically detectable weakness, and clinical examinations are easier, timelier, and more convenient to perform than electrophysiology examinations. However, clinical examinations usually cannot be conducted in the early disease course due to suboptimal levels of consciousness or attentiveness. Electrophysiologic studies may have been more sensitive for detecting subclinical ICUAW in both the corticosteroid and control groups, thus resulting in a nonsignificant effect of corticosteroid use on ICUAW in this subgroup. These considerations may represent an alternative explanation for the different outcome.

Our subgroup analyses showed that there was no significant association between the use of corticosteroids and ICUAW in patients with sepsis. Corticosteroids are a critical treatment for patients with sepsis, and the incidence of this condition’s adverse event, ICUAW, was not significantly different in this review. A therapeutic benefit of early low-dose corticosteroid therapy for decreasing mortality was found in septic shock patients with the highest severity of illness [9]. Low-dose and short-term corticosteroid therapy could improve the prognosis of specific critically ill populations without increasing the risk of ICUAW. Our subgroup analyses demonstrated that studies limited to patients with mechanical ventilation still revealed the significant association between corticosteroids and ICUAW. ICUAW significantly increases the duration of mechanical ventilation [2, 38, 39], and thus the benefits of corticosteroids should be weighed against the adverse effect in ICUAW. Our subgroup analyses revealed that studies limited to relatively large sample sizes still demonstrated a significant association between corticosteroid use and ICUAW, and this result partly demonstrates the stability of the overall effect size.

Studies were excluded for the following common reasons: the study design was not a RCT or prospective cohort, insufficient data were reported, and clear diagnostic criteria were lacking. Only RCTs and prospective cohort studies were included in the review. However, only three studies controlled for other additional factors based on multivariate analysis. We demonstrated a modest association between the use of corticosteroids and ICUAW, without adjustment for potential confounders.

There are limitations to our review. The included studies were not population-based cohort studies. Temporal trends were not examined in the included studies. Baseline exposure to corticosteroids was not reported in the included studies and thus could not be examined via meta-regression. Because different risk factors existed across the included studies and because few studies were designed to adjust for other independent risk factors, primary analysis was performed using a univariate approach without adjustment for potential confounders. High levels of heterogeneity were identified for all of the outcomes. We analyzed the outcomes in subgroups classified by study design, diagnostic methods, sample sizes, and study participants in an effort to reduce methodological and clinical heterogeneity; however, substantial statistical heterogeneity remained despite these attempts. Therefore, a random effects model rather than a fixed effects model was selected to address the observed heterogeneity. Additionally, none of the included prospective cohort studies reported the degree of missing data and how missing data were processed, and thus only a form of per-protocol analysis was performed.

## Conclusion

First, our review demonstrates a statistically significant association between corticosteroid use and ICUAW. Clinicians should limit exposure to corticosteroids or shorten the administration time to decrease the incidence of ICUAW. Second, we did not find a significant association between the use of corticosteroids and ICUAW in patients with sepsis. Third, our review suggests a significant association between corticosteroid use and ICUAW in patients with mechanical ventilation. For specific critically ill patients, clinicians should target low-dose and short-term corticosteroid therapy in clinical practice to limit the adverse effects of the drugs. Future research should focus on RCTs or prospective cohort studies by performing multivariable adjustment for confounders to identify the associations between the use, duration, and total doses of corticosteroids and ICUAW.

## Additional files

Additional file 1: PubMed search strategy. (DOCX 15 kb)

Additional file 2: Summary of findings for the main comparison. (DOCX 14 kb)

## Abbreviations

ARDS: Acute respiratory distress syndrome
CI: Confidence interval
GRADE: Grading of Recommendations, Assessment, Development and Evaluation
ICU: Intensive care unit
ICUAW: Intensive care unit-acquired weakness
MRC: Medical Research Council
OR: Odds ratio
RCT: Randomized controlled trial
SIRS: Systemic inflammatory response syndrome
