# Supplementary material for: Corticosteroid use and intensive care unit-acquired weakness: a systematic review and meta-analysis
Source: Crit Care. 2018 Aug 3;22:187. doi: 10.1186/s13054-018-2111-0 (PMC6091087; doi:10.1186/s13054-018-2111-0)
Supplement: Supplementary file 1 — PubMed search strategy. (DOCX 15 kb) [file 13054_2018_2111_MOESM1_ESM.docx]

Additional file **1-Search Strategy PubMed**

**Domain: ICU patients**

(((((((((((critical care) OR ICU) OR intensive care) OR critical ill) OR critical illness) OR critically ill)) OR ((("Intensive Care Units"[Mesh]) OR "Critical Care"[Mesh]) OR "Critical Illness"[Mesh])))

**Determinant：Corticosteroid**

(((("Steroids"[Mesh]) OR "Glucocorticoids"[Mesh]) OR "Adrenal Cortex Hormones"[Mesh])) OR ((((((Corticosteroid) OR steroid) OR glucocorticoid) OR Corticosteroids) OR steroids) OR glucocorticoids)

**Outcome: Weakness**

((((((((((((("Muscular Diseases"[Mesh]) OR "Muscle Weakness"[Mesh]) OR "Polyneuropathies"[Mesh]) OR "Paralysis"[Mesh]) OR "Paresis"[Mesh]) OR "Neuromuscular Diseases"[Mesh])) OR ((((((((((((((((((paralysis) OR paresis) OR quadriplegia) OR weakness) OR muscular disease) OR muscular diseases) OR neuromuscular disease) OR neuromuscular diseases) OR myopathy) OR myopathies) OR neuropathy) OR neuropathies) OR polyneuropathy) OR polyneuropathies) OR polyneuromyopathy) OR polyneuromyopathies) OR neuromyopathy) OR neuromyopathies)) OR (((((((CIM) OR CIP) OR CIPM) OR CIPNM) OR ICUAW)) OR ICUAP))) OR CINMA))
