# Supplementary material for: Corticosteroid use and intensive care unit-acquired weakness: a systematic review and meta-analysis
Source: Crit Care. 2018 Aug 3;22:187. doi: 10.1186/s13054-018-2111-0 (PMC6091087; doi:10.1186/s13054-018-2111-0)
Supplement: Supplementary file 2 — Summary of findings for the main comparison. (DOCX 14 kb) [file 13054_2018_2111_MOESM2_ESM.docx]

Additional file 2- Summary of findings for the main comparison

| **Corticosteroid Use and Intensive Care Unit-Acquired Weakness** | | | | | | |
| --- | --- | --- | --- | --- | --- | --- |
| **Patient or population:** ICU patients **Settings:** ICU **Intervention:** Corticosteroid Use **Comparison:** Control | | | | | | |
| **Outcomes** | **Illustrative comparative risks* (95% CI)** | | **Relative effect (95% CI)** | **No of Participants (studies)** | **Quality of the evidence (GRADE)** | **Comments** |
|  | Assumed risk | Corresponding risk |  |  |  |  |
|  | **Control** | **Corticosteroid Use** |  |  |  |  |
| **Incidence of ICUAW** | **190 per 1000** | **248 per 1000** (167 to 350) | **OR 1.40**  (0.85 to 2.29) | 375 (1 RCT) | ⊕⊕⊕⊕ **high** |  |
| **Incidence of ICUAW** | **364 per 1000** | **521 per 1000** (417 to 623) | **OR 1.90**  (1.25 to 2.89) | 2012 (17 prospective cohort studies) | ⊕⊝⊝⊝ **very low**^1^ |  |
| *The basis for the **assumed risk** (e.g. the median control group risk across studies) is provided in footnotes. The **corresponding risk** (and its 95% confidence interval) is based on the assumed risk in the comparison group and the **relative effect** of the intervention (and its 95% CI). **ICU:** Intensive care unit; **ICUAW:** Intensive care unit-acquired weakness; **CI:** Confidence interval; **OR:** Odds ratio; RCT: Randomized controlled trials; | | | | | | |
| GRADE Working Group grades of evidence **High quality:** Further research is very unlikely to change our confidence in the estimate of effect.  **Moderate quality:** Further research is likely to have an important impact on our confidence in the estimate of effect and may change the estimate. **Low quality:** Further research is very likely to have an important impact on our confidence in the estimate of effect and is likely to change the estimate. **Very low quality:** We are very uncertain about the estimate. | | | | | | |
| ^1^ Downgraded three levels due to risk of bias (loss of missing data and intention to treat analysis) and serious inconsistency (I² = 69%). | | | | | | |
